# Raman Imaging of Pathogenic *Candida auris*: Visualization of Structural Characteristics and Machine-Learning Identification

**DOI:** 10.3389/fmicb.2021.769597

**Published:** 2021-11-12

**Authors:** Giuseppe Pezzotti, Miyuki Kobara, Tenma Asai, Tamaki Nakaya, Nao Miyamoto, Tetsuya Adachi, Toshiro Yamamoto, Narisato Kanamura, Eriko Ohgitani, Elia Marin, Wenliang Zhu, Ichiro Nishimura, Osam Mazda, Tetsuo Nakata, Koichi Makimura

**Affiliations:** ^1^Ceramic Physics Laboratory, Kyoto Institute of Technology, Kyoto, Japan; ^2^Department of Immunology, Graduate School of Medical Science, Kyoto Prefectural University of Medicine, Kyoto, Japan; ^3^Department of Orthopedic Surgery, Tokyo Medical University, Shinjuku-ku, Tokyo, Japan; ^4^Department of Dental Medicine, Graduate School of Medical Science, Kyoto Prefectural University of Medicine, Kyoto, Japan; ^5^The Center for Advanced Medical Engineering and Informatics, Osaka University, Suita, Osaka, Japan; ^6^Division of Pathological Science, Department of Clinical Pharmacology, Kyoto Pharmaceutical University, Kyoto, Japan; ^7^Division of Advanced Prosthodontics, The Jane and Jerry Weintraub Center for Reconstructive Biotechnology, UCLA School of Dentistry, Los Angeles, CA, United States; ^8^Medical Mycology, Graduate School of Medicine, Teikyo University, Itabashi-ku, Tokyo, Japan

**Keywords:** Raman imaging, Raman spectroscopy, *Candida auris*, machine-learning, glucans, ergosterol

## Abstract

Invasive fungal infections caused by yeasts of the genus *Candida* carry high morbidity and cause systemic infections with high mortality rate in both immunocompetent and immunosuppressed patients. Resistance rates against antifungal drugs vary among *Candida* species, the most concerning specie being *Candida auris*, which exhibits resistance to all major classes of available antifungal drugs. The presently available identification methods for *Candida* species face a severe trade-off between testing speed and accuracy. Here, we propose and validate a machine-learning approach adapted to Raman spectroscopy as a rapid, precise, and labor-efficient method of clinical microbiology for *C. auris* identification and drug efficacy assessments. This paper demonstrates that the combination of Raman spectroscopy and machine learning analyses can provide an insightful and flexible mycology diagnostic tool, easily applicable on-site in the clinical environment.

## Introduction

Invasive candidiasis, a life-threatening opportunistic infection, nowadays represents a major cause of morbidity and mortality (Méan et al., 2008). In this pathological context, the emergence of multidrug species in the genus *Candida* is spread worldwide and poses severe public health implications. *Candida albicans* has long been the predominant species, but a recent shift has occurred toward non-*albicans* species with reduced susceptibility to antifungal agents. *Candida auris* was first identified and classified in the Makimura’s research group in 2009 (Sato et al., 2009). The multidrug-resistant *C. auris* resists commonly used drugs and often eludes a correct identification (Chaabane et al., 2019). In a lack of prompt and reliable microbiological identifications, the spread of infection by *C. auris* is unpredictable, and the situation is further exacerbated by unreported cases. Achieving an appropriate management of invasive candidiasis by *C. auris* entirely depends on two distinct factors: a rapid identification of species and a timely selection of an effective drug.

Speciation of *Candida* species can be performed by a number of different techniques, which are classified into two main categories: phenotypic/biochemical and molecular tests. Tests belonging to the former category, i.e., germ tube test, chlamydospore formation test, sugar assimilation, and sugar fermentation tests, commonly exploit directly observable characteristics. The germ tube test is the fastest method, but it only provides a limited range of speciation (*C. albicans* and *Candida dubliniensis* vs. all other *Candida* species) and is prone to false positive results (Crist et al., 1996). In the case of identification failure by the germ tube test, chlamydospore formation test or sugar assimilation and fermentation tests are usually performed. However, these methods are labor intensive (although recently being conspicuously automatized) and time consuming (3–14 days) (Chander, 2009). Newer commercially available tests, which include CHROMagar^TM^, Candida Plus (Mulet Bayona et al., 2020; Borman et al., 2021), API systems, and Vitek 2 ID system, offer more rapid and/or cost effective choices. However, none of the above-mentioned tests is capable to differentiate *C. auris* from other *Candida* yeast species (CDC, 2019). On the other hand, molecular tests specifically targeting drug-resistant *C. auris* have been developed, which are based on quantitative polymerase chain reaction (qPCR), real-time polymerase chain reaction (PCR), or loop-mediated isothermal amplification (LAMP) (Yamamoto et al., 2018; Camp et al., 2020). The assay time for these tests is in the order of 1 to few hours, excluding the time required for DNA extraction. Therefore, they are quite time consuming, despite being highly reliable. As a separate type of approach, the matrix-assisted laser desorption/ionization time of flight mass spectrometry (MALDI-TOF MS) method has the unique advantage of enabling the analysis from aliquots of positive blood culture bottles after a short time needed for protein extraction. MALDI-TOF MS analyses require times in the order of 90s to identify the fungus developed on the surface of a solid. However, detections from biological fluids (i.e., from positive haemoculture bottles) might take about 30 min. The main advantage of this method is its capacity of identifying all pathogens included in the database, including *C. auris* (Bal and McGill, 2018; Camp et al., 2020). Methods of genomic analysis have revealed fundamental aspects of *Candida* species and opened the path to gene deletion technologies. These technologies have been adapted to track *C. auris* and to screen it for drug resistance, mating, and virulence (Grahl et al., 2017). Among these methods, the clustered regularly interspaced short palindromic repeat (CRISPR)-Cas9 genome modification systems, which was originally expression-constructed for *C. albicans* species, has recently been modified to enable genetic analyses of *C. auris* (Munoz et al., 2018). Precisely sequencing genomes from different *Candida* clades and species requires access to genomic sequence data with long lists of genes taking significant time to be analyzed (Skrzypek et al., 2018). Overall, the presently available identification methods for *Candida* species face a severe trade-off between testing speed and accuracy.

Different from genomics, Raman spectroscopy follows a structural analytical path to identify different yeast species. Resembling a molecular fingerprint, the Raman spectrum provides highly specific chemical information without requiring exogenous probes. Combined with high-resolution hyperspectral imaging techniques, Raman spectroscopy can be used as a molecular microscopy tool that enables *in situ* label-free screening of yeast cells (Noothalapati et al., 2016). The availability of a large number of spectra from Raman imaging then provides the opportunity to apply chemometric methods to yeast speciation. Chemometrics coupled with Raman analyses and imaging has so far been successfully employed to screen the quality of pharmaceutical products (Neuberger and Neususs, 2015), to unfold polymorphic transitions (Farias and Carneiro, 2014), to discriminate oral (Zhu et al., 2007) and pathogenic bacteria (Ho et al., 2019), and to identify biological contamination (Roesch et al., 2005a). Raman spectroscopy has already been applied to characterize *Candida* isolates and related biofilms in order to link the observed Raman spectroscopic characteristics to their genomic diversity (Samek et al., 2014; Chouthai et al., 2015; Potocki et al., 2019; Rebrosova et al., 2019); yet, such analyses have not been performed on multidrug-resistant *C. auris* isolates. Here, we present a preliminary study that combines Raman spectroscopy and imaging with chemometrics to develop a rapid and accurate algorithm for differentiating *C. albicans* from *C. auris*. Despite the presented Raman analyses being limited to only two (out of five known) *C. auris* clades, they suggest that the presented Raman platform might allow for a fast and accurate identification of *C. auris* species within minutes, thus potentially reducing healthcare costs and antibiotic misuse.

## Experimental Procedures

### *Candida* Species

*Candida albicans* ATCC^®^ 90028 (*C. albicans*) cells were purchased from the American Type Culture Collection (ATCC; Manassas, VA, United States). The following *C. auris* isolates were used: the LSEM 3673 (belonging to the South African Clade III) and the LSEM 0643 (or JCM15448T; belonging to the East Asian Clade II). The two latter clades were provided by Teikyo University. A brain heart infusion (BHI) medium was prepared by adding 25 g of BHI broth (Nihon Pharmaceutical Co., Ltd., Tokyo, Japan) into 1 L of distilled water and successively boiling the mixture. After sterilizing in an autoclave at 121°C for 15 min, the mixture was poured into 35 mm glass bottom dish (MatTek Life Sciences, MA, United States). The *C. auris* clades were cultured in SD agar at 36°C for 24 h under atmospheric pressure before Raman spectroscopic measurements. The *C. albicans* clade was cultured in RPMI 1640 Media (Nacalai Tesque, Inc., Kyoto, Japan) supplemented with 10% FCS for 24 h at 37°C in 35 glass bottom dishes.

### *In situ* Raman Spectroscopy

Raman spectra were collected *in situ* on as-cultured single isolate *C. albicans* and two isolates *C. auris* (Clades II and III) living yeast cells. Spectra were obtained using a dedicated instrument (LabRAM HR800, Horiba/Jobin-Yvon, Kyoto, Japan) operating with a 20× optical lens with the spectroscope set in confocal mode. The spectroscope was equipped with a holographic notch filter enabling efficient and high spectrally resolved acquisitions. Excitation was made with a 532 nm solid-state laser source operating at 10 mW. A spectral resolution of better than 1 cm^–1^ was achieved by using an internal reference (neon emission) to calibrate the spectrometer. The Raman scattered light was monitored by a single monochromator connected with an air-cooled charge-coupled device (CCD) detector (Andor DV420-OE322; 1024 × 256 pixels). The acquisition time for a single spectrum was typically 10 s. Thirty spectra were collected at different locations over an area of ∼2 mm^2^ for each clade and averaged in order to obtain a representative spectrum for each clade.

Reference Raman spectra were collected on pure compounds and stored in a purposely built library. The reference library compilation contained more than 40 elementary compounds (simply referred to as the “library,” henceforth), including polysaccharides (e.g., chitin, β-1,3-glucans, β-1,6-glucans, and α-1, 3-glucans), mono- and disaccharides (e.g., trehalose, β-D-glucose, D-dextrose), lipids (e.g., triolein, trilinolein, 1,2-dipalmitoyl-L-α-lecithin), polyols [e.g., D-(+)-arabitol and L-(−)-arabitol], and other key molecules such as adenine, ergosterol, and glycine. The spectra from the pure compounds were collected with a highly resolved spectrometer (T-64000; Jobin-Ivon/Horiba Group, Kyoto, Japan) equipped with a nitrogen-cooled CCD detector (CCD-3500V, Jobin-Ivon/Horiba Group, Kyoto, Japan). The excitation source in these latter experiments was a 514 nm line of an Ar-ion laser operating with a nominal power of 200 mW. The spectral resolution was better than 1 cm^–1^.

Raman imaging of *C. albicans* cells was obtained using a dedicated Raman device (RAMANtouch, Nanophoton Co., Minoo, Osaka, Japan) operated in microscopic measurement mode with confocal imaging capability in two dimensions. This Raman microscope can achieve ultra-fast simultaneous image acquisition of up to 400 spectra. The spectroscope was specially designed to be compatible with cells’ life. It used an excitation source of 532 nm. The spectral resolution was ∼2 cm^–1^ (spectral pixel resolution equal to 0.3 cm^–1^/pixel) with an accuracy in laser spot location of 100 nm. Raman hyperspectral images were generated using commercially available software (Raman Viewer, Nanophoton Co., Minoo, Osaka, Japan). With the purpose of avoiding possible distortions, the Raman images were generated using intensity ratios in the normalized spectra. In order to minimize errors related to spectral resolution and possible shifts in band position, we used the average intensity of the pixels at the band nominal location ±3 pixels, rather than single pixel intensity.

### Machine Learning Algorithm for Spectral Deconvolution

The experimentally obtained Raman spectra were preliminary treated with a baseline subtraction procedure and then automatically deconvoluted into a series of Voigtian sub-bands. Baseline subtraction and deconvolution procedure were performed using options available in commercial software (LabSpec 4.02, Horiba/Jobin-Yvon, Kyoto, Japan). The software applied a polynomial-fitting criterion for baseline subtraction. All spectra were analyzed for their relative intensity after normalization to the glucose ring signal (seen at 478 cm^–1^). The average spectra, *S*_*av*_(ν), was then fitted with an automatic solver, which exploited a linear polynomial expression of Voigtian functions, *V*(Δν, σ, γ); with ν, Δν, σ, and γ representing the Raman frequency, the shift in frequency from each sub-band’s maximum (ν_0_), the standard deviation of each Gaussian component, and the full-width at half-maximum of the Lorentzian component, respectively. A working algorithm was applied to match the experimental spectrum upon searching for the minimum value of the following equation:


(1) $${\text{S}}_{a\,v}\,\left(\nu \right)-\Sigma_{i}\,\alpha_{i}\,\Sigma_{j}\,\beta_{\mathrm{ij}}\,{\text{V}}_{\mathrm{ij}}\,\left(\nu_{0},\Delta \,\nu ,\sigma ,\gamma \right)≅0$$

where the index i locates each compound in a series of n contributing to the overall spectrum, and the index j locates the Voigtian sub-bands of a series of m in the Raman spectrum of each compound of an n series. A computer program was built up for locating a selected series of Voigtian sub-bands from pre-selected compounds belonging to the library, including mono-, di-, and polysaccharides, specific lipids, polyols, and other key molecules, pre-selected according to previously published literature on the structure of *Candida* species. Upon operating a pre-selection of the component molecules from the library, the algorithm located the best fit to the experimental spectra. The computational procedure preserved relative intensities (β_*ij*_), spectral positions (ν_0_), and full-width at half-maximum (σ and γ) values for the individual sub-bands of the deconvoluted spectra from each elementary compound (i.e., within ±3 cm^–1^, considering the resolution of the spectrometer and the possibility of slight alterations in molecular structure). The adopted criteria for the selection of band positions, relative intensity, and bandwidths provide a number of mathematical constraints that allow a univocal deconvolution of the experimental spectra. When specific sub-bands were located, which the solver could not fit according to the pre-selected library, the software labeled them as unknown and required adding a new compound from the library in the pre-selection. Then, a search for additional compounds is launched in the library to match the unknown band following the same criteria as described above. Upon adjusting for the overall intensity contribution (α_*i*_) of each elementary compound within the given constraints, the software located a best fit for the experimental spectrum.

The output of this newly developed machine-learning computational program was threefold: (1) it automatically screened the experimental spectra and proposed a deconvolution by best fitting the average spectrum based on Eq. 1; (2) it indicated the main molecules that contributed the observed intensity of each sub-band; and (3) it located sub-bands whose signal intensity was contributed at >90% by a single reference molecule.

Regarding the details of the machine learning algorithm and related training/validation procedures, we used a *K*-means clustering algorithm based on sets of 4 × 104 spectra obtained from Raman imaging using arrays of 100 arrays of 400 spectra. On all investigated samples, the algorithm was able to identify the main cluster related to the presence of different *Candida* species. *C. auris* presented several distinct traits, including that related the ergosterol/D-arabitol peak at ∼715 cm^–1^, as discussed later in the experimental results.

### Chemometric Analysis

Statistical analyses and visualization of the large Raman data sets obtained during high-resolution imaging were performed according to principal component analysis (PCA) (Wold et al., 1987). The PCA analysis was carried out on the Origin software platform (OriginLab^®^ Co., Northampton, MA, United States). PCA enabled summarizing the information with a set of two “summary indices,” referred to as principal components PC1 and PC2, which clearly differentiated the Raman spectra of different *Candida* clades. In the specific case of *Candida* analyses, coupling PCA with a machine-learning algorithm of spectral deconvolution (as shown in the previous section) allowed extraction and interpretation of the maximum relevant chemical information that unequivocally differentiates *Candida* species and helped in understanding their chemical characteristics.

## Experimental Results

### Average Raman Spectra From Different *Candida* Species

**FIGURE 1:**
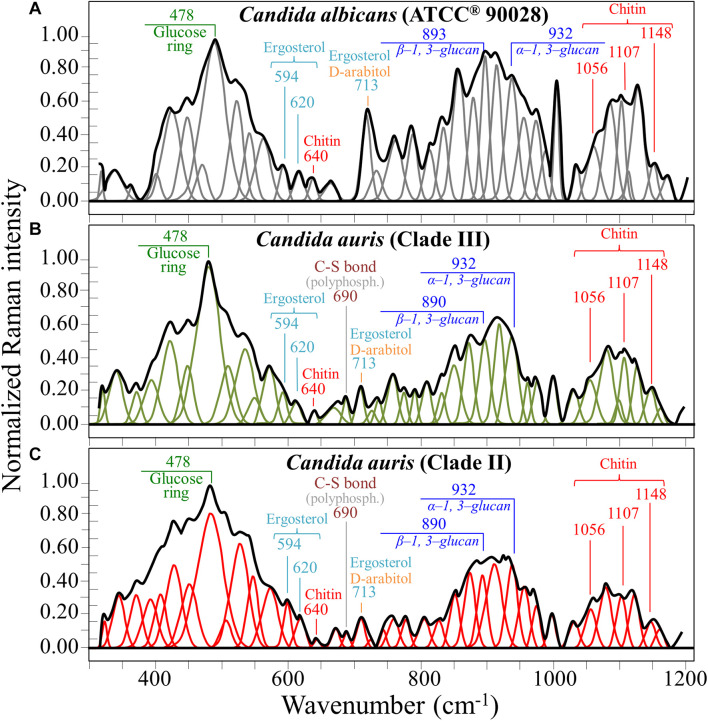
Average Raman spectra of **(A)** *C. albicans* (ATCC^®^ 90028), **(B)** *C. auris* (Clade III), and **(C)** *C. auris* (Clade II) in the wavenumber interval 300–1200 cm^–1^; spectra are normalized to the glucose ring band at ∼478 cm^–1^ and deconvoluted into Voigtian sub-bands by means of the machine-learning algorithm described in section “Chemometric Analysis.” The wavenumbers and the assignments of the main bands discussed in the text are given with labels in inset.

**FIGURE 2:**
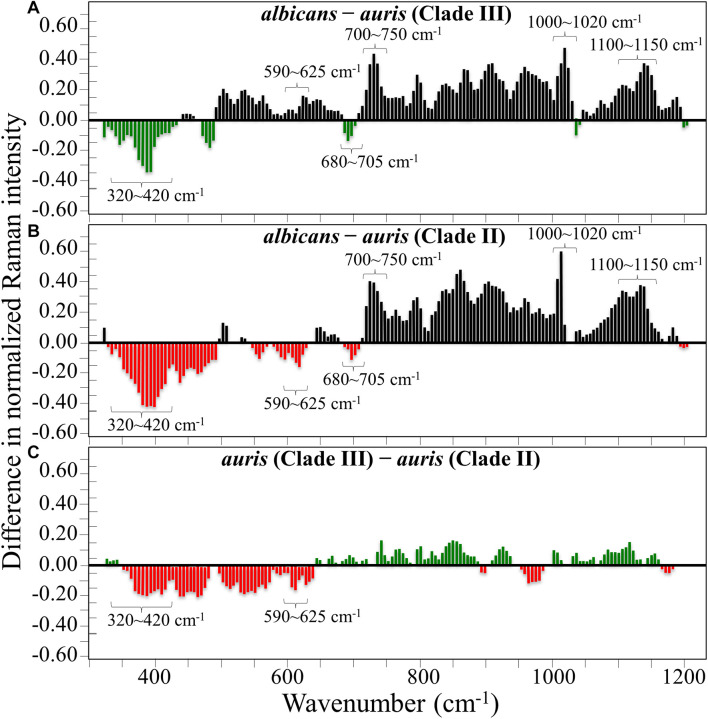
Results of subtraction of the average normalized spectra in Figure 1: **(A)** subtraction of the *C. auris* (Clade III) spectrum from the *C. albicans* spectrum; **(B)** subtraction of the *C. auris* (Clade II) spectrum from the *C. albicans* spectrum; **(C)** subtraction of the *C. auris* (Clade II) spectrum from the *C. auris* (Clade III) spectrum.

**FIGURE 3:**
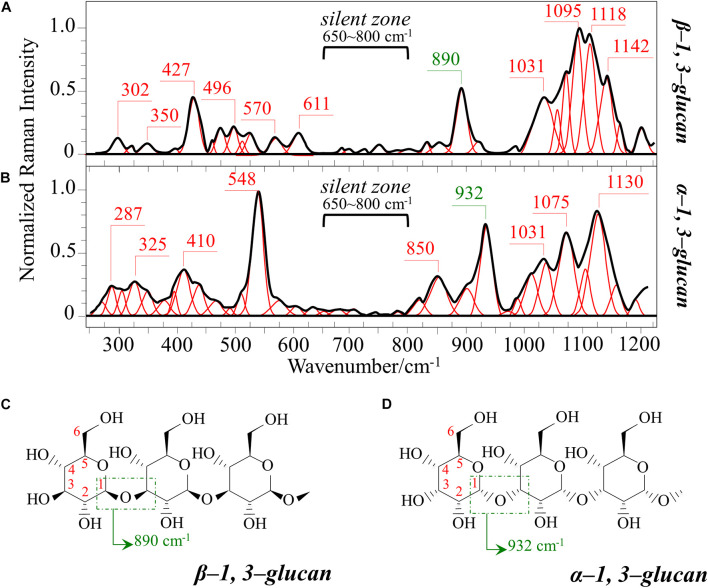
Reference Raman spectra of: **(A)** β-1, 3-glucans and **(B)** α-1, 3-glucans (frequencies in cm^–1^ of the main bands given by labels in inset); **(C,D)** the respective glucan structures (cf. labels in inset). The spectra are deconvoluted in series of Voigtian bands.

**FIGURE 4:**
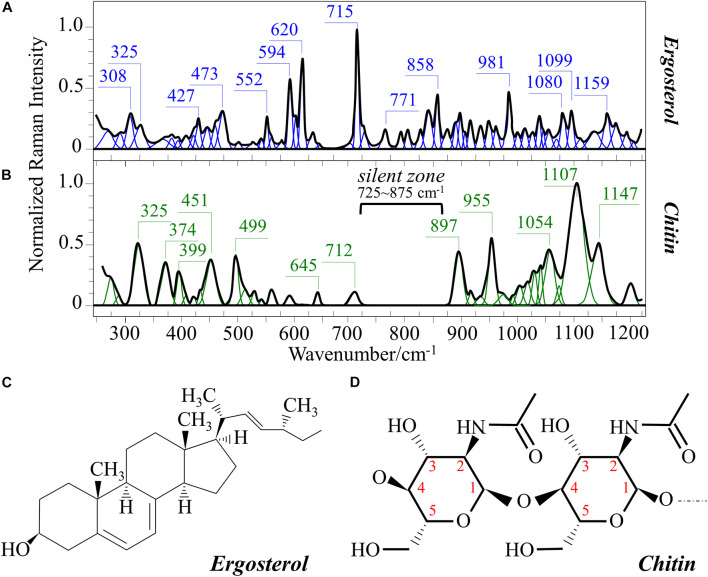
Reference Raman spectra of: **(A)** ergosterol and **(B)** chitin (frequencies in cm^– 1^ of the main bands given in inset); **(C,D)** give the respective molecular structures (cf. labels in inset). The spectra are deconvoluted in series of Voigtian bands.

Figures 1A–C show the average Raman spectra of *C. albicans*, *C. auris* (Clade III), and *C. auris* (Clade II), respectively, as collected in the wavenumber interval 300–1200 cm^–1^. The spectra were first normalized to their common glucose ring band centered at ∼478 cm^–1^ and then deconvoluted into a series of Voigtian sub-bands by means of the machine-learning algorithm described in section “Chemometric Analysis.” The initial choice of molecular components included β-1, 3-glucans, α-1, 3-glucans, chitin, ergosterol, D-arabitol, trehalose, and adenine. A comparison among the collected (average) spectra revealed a number of vibrational bands common to all *Candida* species, but also bold differences in relative intensity and missing vibrational signals. Such differences are important because they locate specific fingerprints that enable the identification of predominant chemical patterns in different species. In an effort to better visualize the differences, spectral subtractions were performed as shown in Figure 2 (cf. labels). Bold differences in band relative intensities were noticed both at low and high wavenumbers. In order to facilitate the interpretation of these differences, reference Raman spectra are shown for four reported main constituents of yeast cells as collected on pure compounds. These spectra belong to a library of Raman spectra, which was built for elementary molecules. Spectra from β-1, 3-glucans and α-1, 3-glucans are shown in Figures 3A,B, respectively (cf. also structures in Figures 3C,D, respectively). Figures 4A–D show reference spectra/structures of ergosterol and chitin, respectively. At the lower measured wavenumbers (i.e., 320–420 cm^–1^), both *C. auris* species showed stronger Raman signals as compared to *C. albicans* (cf. Figures 2A,B). In yeasts, these wavenumbers mainly correspond to ring deformation and skeletal vibrations in glucans and chitin (De Gussem et al., 2005). The most prominent band in this low-wavenumber interval, which is located at ∼440 cm^–1^, included signal contributions from α-1, 3-glucans, chitin, and ergosterol (cf. Figures 3, 4), which were hard to separate. On the other hand, the machine-learning algorithm (i.e., Eq. 1) located two bands (at 594 and 620 cm^–1^) as mainly (>95%) contributed by ergosterol. Upon comparing the sum of the relative intensities of these two bands (cf. Figures 1, 2) in different clades, the highest fraction of ergosterol appeared to occur in *C. auris* (Clade II) (0.50) and the lowest in *C. auris* (Clade III) (0.34), with the *C. albicans* lying in the middle (0.41). Note that also the intense band at ∼713 cm^–1^ is contributed by ergosterol, but it contains additional (non-negligible) contributions from D-arabitol, chitin, and adenine. Accordingly, it could hardly serve as a fingerprint for ergosterol alone. Spectral differences in ergosterol bands are important since it has recently been suggested that mutations in *Candida* species could cause depletion and alteration of the ergosterol composition with an impact on antifungal resistance (Kean and Ramage, 2019). This point will be discussed later.

The wavenumber zone at 680–705 cm^–1^, which was another additional (narrow) interval of stronger Raman intensity for both *C. auris* species (cf. Figure 2), corresponded to a well distinct band centered at ∼690 cm^–1^. This band, which was only present in *C. auris* species (cf. Figure 1), was located by means of the machine-learning algorithm as one requiring the choice of an additional compound from the library, besides the pre-selected components given above. As a matter of fact, in this wavenumber interval, both glucans and chitin present either a silent zone or very weak signals (cf. Figures 3A,B and 4B, respectively), and ergosterol shows no Raman bands (cf. Figure 4A). Therefore, the *C. auris* band centered at ∼690 cm^–1^ should belong to a different compound. One possible candidate in assigning this band are S-containing amino acids, namely, methionine and cysteine, because the C–S bond stretching vibration scatters in this zone (Diaz Fleming et al., 2009). The observed band frequency corresponds to C–S stretching in methionine and/or cysteine (in *gauche* and *trans* configurations, respectively). In favor of this hypothesis is the fact that the Raman intensity is generally strong for bonds involving sulfur even when only small fractions of S-containing amino acids are present. The *C. auris* Clade III displayed a Raman intensity at 690 cm^–1^ 60% higher than the *C. auris* Clade II (cf. Figures 1B,C). In a recent paper by Costa et al. (2015) mechanisms of flucytosine resistance in *Candida glabrata* were found to link with sixteen genes related to nitrogen metabolism. These included genes involved with biosynthesis of methionine (MET2 and MET7), among other genes related to proline, tyrosine/phenylalanine, and leucine productions. Based on this finding, it was suggested a role of methionine fractions included in the external membrane proteins in the drug-resistance behavior of different *Candida* species. In this context, note also that the relative intensity of the Raman band at ∼1002 cm^–1^ (related to phenylalanine ring vibrations) was much more pronounced in the spectrum of *C. albicans* as compared to those of both the investigated *C. auris* isolates (cf. Figures 1, 2). Methionine serves as the major biological methyl donor in methylation reactions, which are essential for the biosynthesis of phospholipids, proteins, DNA, and RNA (Fontecave et al., 2004). Similar to the case of subgingival bacteria (Gosschalk et al., 2020), increased virulence could also be related to S-containing transpeptidase enzymes that covalently attach proteins to peptidoglycan precursors. Bednarova et al. (2012) assigned a 688 cm^–1^ band (symmetric P–O–P stretching) to polyphosphates contained in yeast vacuoles. This band was one of a couple with a threefold stronger band centered at ∼1154 cm^–1^ (symmetric PO_2_ stretching), which was indicated as a marker for vacuole formation. The presence of vacuoles is a sign of adaptation of *C. albicans* to environmental changes due to severe nutrition limitations, and a sign of its reaction against host immune defense (Kim and Sudbery, 2011). The function of vacuoles is to store amino acids, ions, and metabolites that serve in pH homeostasis and osmoregulation, while also playing a crucial role in breaking peptide links and recycling nutrients (Li and Kane, 2009). In the Raman spectra of Figure 1, a band at ∼690 cm^–1^ is completely missing in *C. albicans* (cf. Figure 1A) and only present in the two *C. auris* isolates (Figures 1B,C). A band at 1154 cm^–1^ is also seen in the spectra of both *C. auris* clades (and missing in the *C. albicans* isolate), but only as a weak shoulder. The 1154 cm^–1^ band in the spectrum of polyphosphate pure compound is about three times more intense than that at 690 cm^–1^ (Bednarova et al., 2012), while the intensity of the shoulder band seen at 1154 cm^–1^ in *C. auris* spectra is about a half of the intensity of the 690 cm^–1^ one. Note also that different types of *Candida* cells were analyzed after being cultured in nutritional medium and not in a stress state. Moreover, secretion of phospholipase and protease enzymes in *C. auris* has been reported to be generate at significantly lower levels as compared to *C. albicans* (Larkin et al., 2017). For all the above reasons, we believe that the correct assignment of the 690 cm^–1^ band is C–S bond stretching.

The membrane of *Candida* species includes both α- and β-glucans polysaccharides, which only differ in their C–O–C bond configurations at the anomeric C1 position (cf. Figures 3C,D). Such difference in bond configuration is reflected in fine morphological differences of the Raman spectrum in correspondence of the wavenumber interval 850–950 cm^–1^, which is sensitive to glycosidic linkages (Noothalapati et al., 2016). Note that the overall relative intensity of the average Raman spectra recorded in this spectral zone was clearly higher in *C. albicans* as compared to Clades II and III of *C. auris* (cf. Figures 2A,B). Additional spectral differences arise in the region of C–H equatorial bending vibrations, which are found at 880–910 and 830–870 cm^–1^ in the cases of β- and α-anomer, respectively (Cael et al., 1974; Corbett et al., 1991). Examination of band overlap with other biomolecules revealed that the deconvoluted sub-band at ∼890 cm^–1^ can be taken as a marker for β-1, 3-glucans, while the antisymmetric ring vibration seen at 932 cm^–1^ is a fingerprint for α-1, 3-glucans in *C. auris*. Note that also the signal at ∼850 cm^–1^ is contributed by α-1, 3-glucans, but it strongly overlaps with a main band from tyrosine (in-plane phenol ring vibrations) and cannot easily be singled out. The above choice of the 932 cm^–1^ marker is in line with previously published studies of α-1, 3-glucans (Synytsya et al., 2009; Mikkelsen et al., 2010). According to the above spectral choices, the fractional ratio of α- to β-glucans of the yeast wall structure can be computed for different *Candida* species as: 0.83, 1.03, and 1.09 for *C. albicans*, *C. auris* (Clade III), and *C. auris* (Clade II), respectively. In summary, both *C. auris* clades possessed lower total amounts of glucans as compared to the *C. albicans* isolate, but they were richer in α-1, 3-glucans.

In the Raman spectra of polysaccharides, the region representing C–C and C–O symmetrical stretching corresponds to the interval 950–1200 cm^–1^. Although all polysaccharides show a number of structural similarities (e.g., the silent zone at intermediate frequencies, 725–875 cm^–1^; cf. Figure 4B), distinctions can yet be made in the C–C/C–O spectral region. This is the case of chitin (Figure 4D), which is structurally different due to the presence of an amide group at the C2 position to replace an OH group (De Gussem et al., 2005). Due to this structural feature, the chitin spectrum shows peculiar bands at 955, 1107, and 1149 cm^–1^, in addition to other spectral features at the lower frequencies of 325 and 645 cm^–1^ (cf. Figure 4B). Upon machine-learning examination of the *Candida* spectra in Figure 1, the triplet at 1056, 1107, and 1148 cm^–1^ and the low-frequency band at 640 cm^–1^ were located as non-overlapping chitin bands and indicated as fingerprints for chitin since they conspicuously preserved both relative intensities and band morphology characteristics of the elementary compound. Building upon this automatic indicator, we compared the relative intensities of chitin-related bands (with respect to the 478 cm^–1^ glucose ring band) among different *Candida* clades and found that *C. albicans* was richer in chitin by ∼31 and 50% as compared to Clades III and II *C. auris*, respectively. Since chitin adds rigidity and structural support to the yeast cell walls, *C. albicans* appeared to possess the most rigid cell-wall structure, while *C. auris* Clade II is the most flexible one.

*Candida albicans* cells experience elevated chitin content in their wall as a consequence of an adaptive (remodeling) mechanism to maintain intact (i.e., not permeable) the cell walls and to fix resistance to specific drugs that inhibit synthesis of β-1, 3-glucans (i.e., echinocandins) (Ibe and Munro, 2021). The same chitin-enrichment remodeling effect was also found in *C. auris* species; however, the extent of such effect was significantly less pronounced (Lara-Aguilar et al., 2021). On the other hand, a higher flexibility of yeast cell walls, which is dictated by the interplay between β-1, 3-glucans and chitin, is also a sign of cellular adaptation to environmental change (Munro, 2013), since it confers resistance to osmotic stress (Ene et al., 2015). Regarding the origin of the osmotic stress resistance, the current understanding links it to the circumstance of yeast cells entering the bloodstream and becoming suddenly exposed to glucose. Such sudden exposure imposes to develop a mechanism of osmotic stress resistance (Van Ende et al., 2019). *C. auris* exploits different strategies to resist various kinds of external stress and to promote virulence as compared to *C. albicans* (Day et al., 2018). Raman spectroscopy reveals here large differences in the structure of yeast cell walls between unstressed *C. albicans* and *C. auris* clades, while much less pronounced differences were found between different *C. auris* clades. The detected spectral differences suggest that the wall structures of *C. auris* clades underwent permanent compositional changes to improve cell wall elasticity, notwithstanding the yeast capacity to manipulate the structure of their walls in a dynamic response to environmental perturbations and to display fast structural realignments to increase survival probability following osmotic shock.

### Raman Imaging of Living Yeast Cells

**FIGURE 5:**
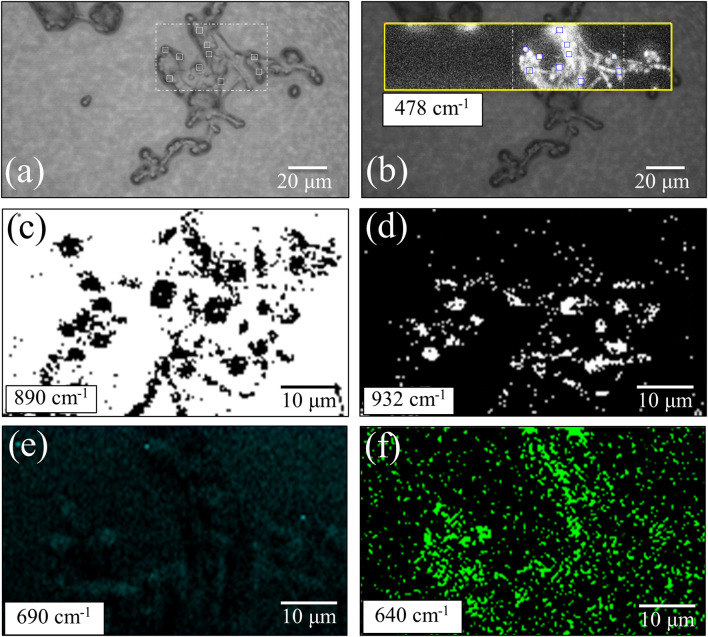
**(a)** Optical micrograph of cultured *C. albicans* cells; spatially resolved Raman maps in: **(b)** glucose rings in membrane polysaccharides at 478 cm^–1^ (in the yellow square region), **(c)** β-1, 3-glucans (in the white broken-line square region) at 890 cm^–1^ (black spots on white background), **(d)** α-1, 3-glucans (in the broken-line square region) at 932 cm^–1^ (white spots on black background), **(e)** S-containing amino acids (in the white broken-line square region) at 690 cm^–1^, and **(f)** chitin at 640 cm^–1^ (in the white broken-line square region). The 10 smaller square areas located in **a,b** represent the regions used for PCA analyses.

**FIGURE 6:**
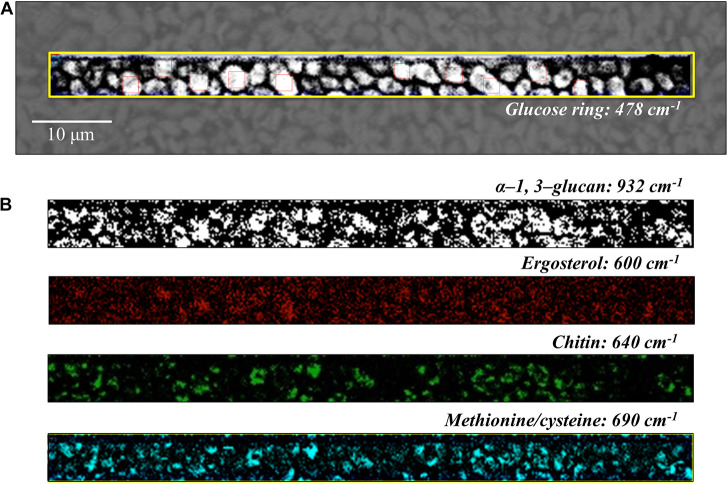
**(A)** Optical micrograph and Raman map of glucose rings in membrane polysaccharides at 478 cm^–1^ (in the yellow square region) of *C. auris* (Clade III) culture; in **(B)** from top to bottom (cf. labels), α-1, 3-glucans at 932 cm^–1^ (white spots on black background), ergosterol at 600 cm^–1^, chitin at 640 cm^–1^, and S-containing amino acids at 690 cm^–1^. The maps in **(B)** correspond to the same square region in **(A)**. The 10 smaller square areas located in **(A)** represent the regions used for PCA analyses.

**FIGURE 7:**
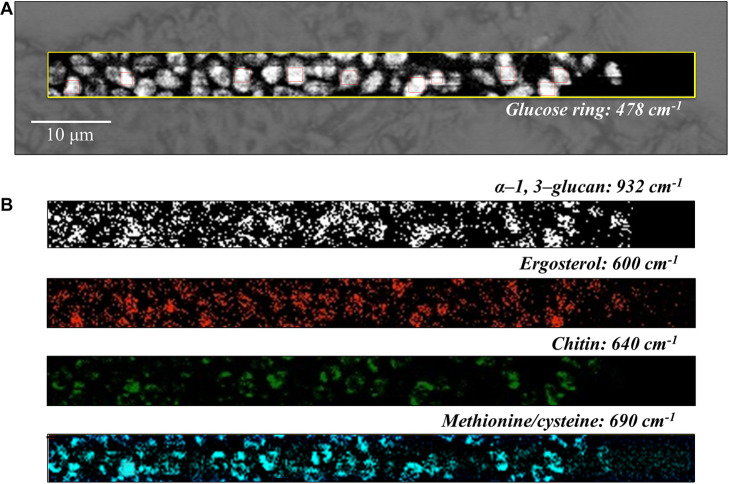
**(A)** Optical micrograph and Raman map of glucose rings in membrane polysaccharides at 478 cm^–1^ (in the yellow square region) of *C. auris* (Clade II) culture; in **(B)** from top to bottom (cf. labels), α-1, 3-glucans at 932 cm^–1^ (white spots on black background), ergosterol at 600 cm^–1^, chitin at 640 cm^–1^, and S-containing amino acids at 690 cm^–1^. The maps in **(B)** correspond to the same square region in **(A)**. The 10 smaller square areas located in **(A)** represent the regions used for PCA analyses.

The machine-learning analyses on average Raman spectra shown in the previous section revealed significant differences in yeast wall composition, including lipids and polysaccharides, and a possible variation in concentration of S-containing amino acids (present both intracellular and in the external membrane proteins). In order to spatially locate the differences and to statistically substantiate the findings, we performed extensive Raman imaging on living cultures of the three investigated *Candida* isolates (the total number of spectra collected per each species is in the order of 10^5^), as shown in Figures 5–7. Figure 5 shows an optical micrograph (a) of *C. albicans* culture and spatially resolved Raman maps (taken in the square region) of signals at 478 cm^–1^ (b), 890 cm^–1^ (c), 932 cm^–1^ (d), 690 cm^–1^ (e), and 640 cm^–1^ (f), representing the entirety of glucose rings in membrane polysaccharides, β-1, 3-glucans, α-1, 3-glucans, S-containing amino acids both intracellular and in membrane mannoproteins, and chitin, respectively. The comparison between the maps of β-1, 3-glucans and α-1, 3-glucans [black spots on white background and white spots on black backgrounds in (c) and (d), respectively] makes quantitative the ratio between these two polysaccharides in the *C. albicans* isolate, which was the richest in β-1, 3-glucans among the investigated species. The fractional ratio computed from black and white spots related to the same area indicated an α/β glucan-ratio ∼0.81, which is in good agreement with the 0.83 value computed from band-intensity ratio in the average spectrum in Figure 1A. Also the conspicuous lack of 690 cm^–1^ signal from C–S bond stretching, already noticed in the average spectrum in Figure 1A, was confirmed in Raman mapping experiments (cf. Figure 5e). The chitin spots in the *C. albicans* map in Figure 5f were quite pronounced in correspondence of the cells, but they also appeared in minor fractions in neighboring areas.

Spatially resolved Raman images of *C. auris* Clades III and II are shown in Figures 6, 7, respectively. Glucose rings in membrane polysaccharides (478 cm^–1^), α-1, 3-glucans (932 cm^–1^), chitin (640 cm^–1^), and S-containing amino acids in membrane mannoproteins (690 cm^–1^) are shown in sections (a) and (b) of each figure (cf. labels). Both *C. auris* clades were confirmed to be richer in α-1, 3-glucans (932 cm^–1^ white spots on black background) and methionine (690 cm^–1^) as compared with *C. albicans*. On the other hand, the ergosterol map showed a detectably higher spot concentration in the *C. auris* Clade II as compared to Clade III (cf. maps at ∼600 cm^–1^ in Figures 6, 7). Finally, the consistency between average spectra and Raman imaging experiments could be confirmed with comparing the chitin maps at ∼640 cm^–1^; Raman imaging confirmed that the *C. auris* Clade II clade was the least chitin rich among the three *Candida* isolates investigated (cf. Figures 5–7).

### Statistical Principal Component Analyses

**FIGURE 8:**
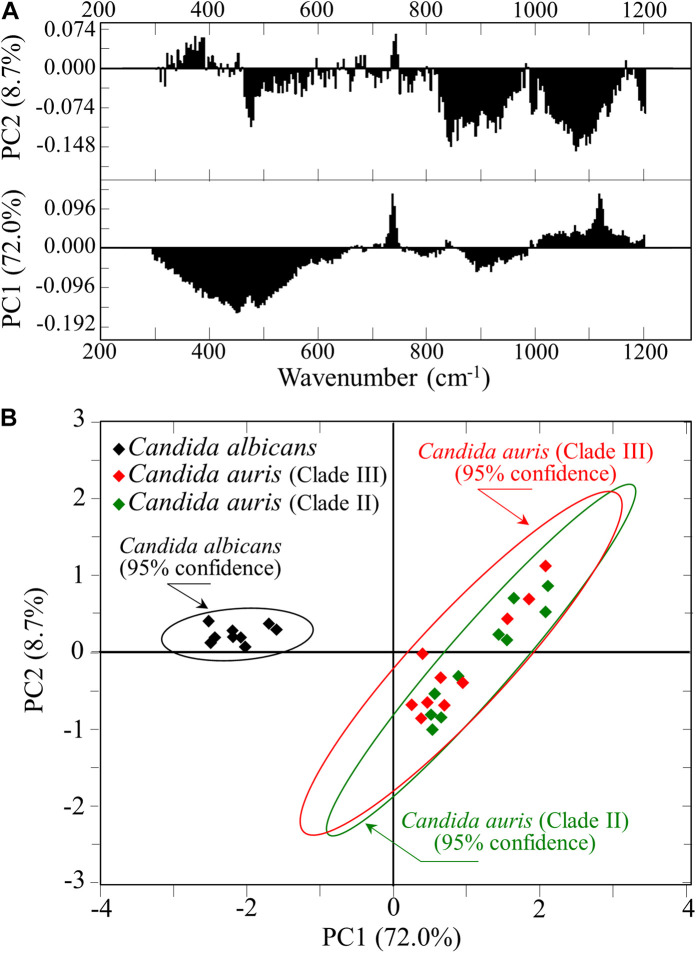
**(A)** First and second principal components (PC1 and PC2, respectively) of a PCA analysis of Raman spectra at the 10 selected locations from maps of *C. albicans* and two *C. auris* clades (Figures 5–7); **(B)** plot of the loading vectors PC1 and PC2 for the spectral region 300–1200 cm^–1^.

For each *Candida* clade investigated, 10 locations were selected as shown by square insets [cf. section (a) of Figures 5–7]. Each location was ∼20 μm^2^ in size and contained 100 spectra, which were in turn averaged to obtain a single spectrum for each location. Figure 8A shows the first and second principal components (PC1 and PC2, respectively) of a PCA analysis conducted on the Raman spectra representative of the above-mentioned 10 selected locations from each map of the three isolates of two *Candida* species investigated. As seen in Figure 8B, *C. albicans* vs. *C. auris* data sets displayed as well separated (95% confidence) upon plotting the loading vectors PC1 and PC2 for the entire spectral region 300–1200 cm^–1^. However, the PCA analysis also pointed out that it was not possible to distinguish between the two *C. auris* clades. The present application of the PCA statistical method, which reduces the dimensionality of the Raman data matrix to only two orthogonal variables, gives clear evidence that it is possible to identify *C. albicans* and *C. auris* by discrimination between their Raman spectra.

As a final observation with reference to Figure 1, one could note that the Raman spectra of *C. albicans* and *C. auris* are markedly different, so that they could be differentiated at a glance even without applying specific algorithms such as machine learning. For example, the relative intensities of the ergosterol peak at ∼713 cm^–1^ or the phenylalanine peak at ∼1000 cm^–1^ could be taken as fingerprints for *C. albicans* with respect to *C. auris* clades. However, besides adding computational exactness and exhaustiveness to the spectral differentiation procedure, the machine-learning algorithm enables to exactly locate the contributions made by different molecular constituents to the structure of different *Candida* species. This approach could ultimately enable quantifying both membrane fluidity/elasticity and antifungal resistance of different mutants.

## Discussion

### Structural Peculiarities of *Candida auris* Revealed by Raman Analyses

The Raman analyses presented in this study have located (with statistical relevance) some fundamental differences in the structures of *C. albicans* and *C. auris*. Specifically, two main differences were related to the polysaccharide structure of the yeast cell membrane, as follows:

(i) Both *C. auris* Clades II and III contained similar fractional ratios of α- to β-glucans, which were 20–30% higher than that measured in *C. albicans*.
(ii) *C. albicans* was richer in chitin by ∼31 and 50% as compared to *C. auris* Clades III and II, respectively.

The two above characteristics suggest that both *C. auris* clades possess cell walls less permeable to external agents and structurally more flexible. α-1, 3-glucans are insoluble in water due to the presence of strong hydrogen bonds, and their accumulation in the yeast cell walls was shown to protect the yeast cells from antifungal agents and degrading enzymes encountered during infection (Fujikawa et al., 2009). A wall structure “engineered” with larger amounts of α-1, 3-glucans could be linked to the fact that the majority of clinical *C. auris* species display resistance to the main classes of antifungals (azoles, polyenes, or echinocandins) (Cernakova et al., 2021). The observed higher fractions of α-1, 3-glucans in both studied *C. auris* clades also likely relate to their reported strong biofilm-mediated resistance (Kean and Ramage, 2019). Note also that the α-1, 3-glucans are more abundant in pathogenic yeasts, and are reported to be needed for their normal morphology and virulence (Ruiz-Herrera and Ortiz-Castellanos, 2019). Regarding the resistance of *C. auris* to osmotic stress, this characteristic is related to a high flexibility of the yeast cell walls. This, in turn, is related to the lower fraction of chitin contained in the cell walls. According to Ene et al. (2015) the β-glucan/chitin interlinked composition of the yeast cell walls inner matrix is key in modulating elasticity and strength. The ability of *Candida* species to display rapid structural realignments in the compositional fractions of glucans vs. chitin greatly impacts survivorship following osmotic shock. *C. auris* was found clearly more resistant to the oxidative stress imposed by H_2_O_2_ than *C. albicans*, and displayed higher levels of resistance to the cationic stress imposed by either sodium or calcium chloride, but it also proved much less capable to adapt to acidic or alkaline pH environments than *C. albicans* (Day et al., 2018). Conversely, *C. albicans* was found capable to undergo cell wall remodeling in acidic environment, which resulted in a promptly enhanced chitin contents (Sherrington et al., 2017). All these specific characteristics, which relate to both composition and (chemical vs. mechanical) resistance of the yeast cell walls, illustrate the unique stress resistance profile of *C. auris* as compared to other *Candida* species. As a matter of fact, the trade off between cell-wall flexibility and chemical stability depends on the type of polysaccharides.

Regarding the structure of cell-wall lipids, the Raman experiments revealed that the *C. auris* Clade II possessed the highest fraction of ergosterol while the *C. auris* Clade III the lowest. It has been recently clarified that mutations in *Candida* species can cause depletion and alteration of the ergosterol composition (Kean and Ramage, 2019). Alterations in the ergosterol pathway, which in turn depend on ergosterol gene mutations, are considered the main responsible for resistance to amphotericin B (Kordalewska et al., 2018). Ergosterol gene mutations lead to reduced ergosterol levels in the yeast plasma membrane, which in turn increases the resistance to polyenes because polyene-based drugs specifically target ergosterol by binding, to form pores or simply sequestrating it to induce membrane disruption (Mesa-Arango et al., 2012; Frias-De-Leon et al., 2020).

An additional spectral difference among the three studied *Candida* isolates was the Raman intensity at ∼690 cm^–1^, which was assigned to C–S bond vibrations in specific rotamers of S-containing amino acids (i.e., methionine and cysteine). At this specific frequency, the *C. auris* Clade III displayed a Raman signal 60% higher than the *C. auris* Clade II, while no signal could be detected in the *C. albicans* species. Methionine affects morphogenesis and virulence through the protein kinase A, a pathway that is also involved in cellular processes related to cell wall integrity and drug sensitivity (Biswas et al., 2007; Schrevens et al., 2018; Kuplinska and Rzad, 2021). Both methionine and cysteine are also important biofilm inducers (Schrevens et al., 2018). *C. albicans* was found capable to synthesize methionine (Prasannan et al., 2009) and to interconvert cysteine and methionine through the trans-sulfuration pathway (Thomas and Surdin-Kerjan, 1997; Schrevens et al., 2018). Therefore, the lack of 690 cm^–1^ Raman signal in the *C. albicans* spectrum can only be attributed to one specific missing rotamer or to definite physiological circumstances of the cultured yeast cells, rather than to an intrinsic structural characteristic.

### Importance of the Raman Approach to *Candida* Species Differentiation

The Raman method is widely applied in cell biology and microbiology with several different purposes, including identification of different types of cell (e.g., cancer and stem cells) (Chan et al., 2006; Kast et al., 2008; Leslie et al., 2012), differentiation between Gram-positive and Gram-negative bacteria (Paret et al., 2010), tracking down of mutant viruses (Yeh et al., 2020), and characterization of environmentally and physiologically driven changes in cellular and microbial metabolism (Pezzotti, 2021; Pezzotti et al., 2021a,b). Raman molecular fingerprints have also been used for the identification of yeasts and fungi (Roesch et al., 2005b). In this study, we have applied machine-learning-supported Raman method and succeeded in differentiating between *C. albicans* and *C. auris* clades. From a general viewpoint, the present findings are important because they show that creating a reference Raman library for clinically important *Candida* pathogens may provide a tool for rapid identification of pathogens in clinical practice. For the specific case studied here, the possibility of locating the presence of *C. auris* clades with a conspicuously reduced identification time could allow a prompt selection of the most appropriate cure. In line with the present state of the art in deep learning applied to biospectroscopy and biospectral imaging (He et al., 2021), we have proposed here a reliable and versatile Raman method to locate subtle variations and hidden features within big data collected on different *Candida* clades. Once translated into clinical practice, the speed of the presented Raman approach will provide a significant advantage for all those patients at immunological risk, who have limited ability to fight invasive fungal infections.

In this context, it is important to note that the MALDI-TOF MS method is already replacing traditional methods for identifying pathogens (including yeasts and fungi) in clinical practice (Rychert, 2019). This technique, which relies on measuring microbial proteins for discriminating different species, can already exploit a wide database; it has also been shown to be faster and more accurate than conventional biochemical methods for definitive identifications of *Candida* species (Bal and McGill, 2018; Camp et al., 2020). The Raman spectroscopic method, which we newly apply here to *C. auris*, is also a suitable tool for fast on-site identifications of *Candida* species. It cannot yet rely on a complete database, but it has a tangible advantage with respect to the MALDI-TOF MS method: it concurrently gives precise information on polysaccharides, protein, lipids, and DNA structures, with respect to their fractions and molecular symmetry. While exploiting this peculiarity, the Raman method can be applied in time-lapse mapping experiments on living cells to follow up in real time the metabolic reactions of the *Candida* species to environmental stress. The Raman findings could become clinically important to obtain molecular-scale evidences in drug development as, for example, in understanding why echinocandin and amphotericin B treatments frequently lead to therapeutic failures among patients suffering from invasive *C. auris* infections. Such *in situ* studies are presently ongoing.

### Limitations of This Study

At the present time, five different *C. auris* clades have been discovered. However, the present basic study yet addresses Raman experiments on only one isolate each from South African and East Asian clades. Accordingly, the present results do not allow us to unequivocally conclude that the presented Raman approach will be valid in general. While it has worked well in speciation of *C. albicans* and *C. auris*, additional work is needed to check whether misidentifications could occur when other *Candida* species are also considered. It should also be noted that different *C. auris* clades possess different degrees of virulence (Borman et al., 2016; Forgacs et al., 2020) and different *in vitro* susceptibility toward different antifungal agents (Szekely et al., 2019; Kovacs et al., 2021; Papp et al., 2021). Therefore, characterizations of all clades are mandatory to confirm whether or not the present Raman spectroscopic approach could be extended to and give unequivocal molecular-scale information about all other *C. auris* clades. Encouraging results were obtained and we are presently completing a number of additional Raman characterizations on different clades/isolates. The results will be published in a forthcoming report. Moreover, samples taken on-site from patients should also be examined in future experiments in order to test the actual suitability of Raman classifications in the clinical practice.

## Conclusion

Differences in Raman fingerprints of *C. albicans* and *C. auris* yeasts belonging to different clades were studies using a double approach based on the analysis of average spectra collected with a resolution 20× and maps of 10^5^ spectra collected with high spatial resolution. The former approach unfolded several spectral signatures traceable to specific structural features peculiar to different *Candida* species. The latter approach, while confirming the characteristics retrieved from average spectra, allowed the application of a chemometric method based on PCA to mechanistically distinguish between *C. albicans* and *C. auris*. We succeeded in unequivocally distinguishing between these two different types of *Candida* yeasts, the proposed identification procedure accomplishing both accuracy and testing speed. Moreover, the results we obtained in classification of different *Candida* species by using machine learning analyses of Raman spectroscopic data are relevant to practical treatments because they reveal structural details that might relate to both virulence and drug resistance characteristics. Future experiments should be conducted to provide assessments of a larger number of *Candida* species in order to build up a comprehensive library. The present findings justify the design and development of a rapid, low-cost, portable Raman system for application as a precise, fast, and reliable diagnostic tool in the clinical practice.

## Data Availability Statement

The raw data supporting the conclusions of this article will be made available by the authors, without undue reservation.

## Author Contributions

GP, TY, NK, OM, and IN contributed to conception and design of the study. MK, TAs, TaN, NM, TAd, and EO performed the analyses. WZ organized the database. EM performed the statistical analysis. GP wrote the first draft of the manuscript. TeN and KM wrote sections of the draft. All authors contributed to manuscript revision, read, and approved the submitted version.

## Conflict of Interest

The authors declare that the research was conducted in the absence of any commercial or financial relationships that could be construed as a potential conflict of interest.

## Publisher’s Note

All claims expressed in this article are solely those of the authors and do not necessarily represent those of their affiliated organizations, or those of the publisher, the editors and the reviewers. Any product that may be evaluated in this article, or claim that may be made by its manufacturer, is not guaranteed or endorsed by the publisher.
